# The minimal clinically important difference for the patient activation measure in a culturally and linguistically diverse cohort with chronic conditions

**DOI:** 10.1007/s11136-026-04197-w

**Published:** 2026-03-01

**Authors:** Robert Boland, Joseph Descallar, Justine M. Naylor, Gavin Williams, Bernadette Brady

**Affiliations:** 1 Discipline of Physiotherapy, Sydney School of Health Sciences, Faculty of Medicine and Health, The University of Sydney, Sydney, NSW Australia; 2 Physiotherapy Department, Fairfield Hospital, Sydney, NSW Australia; 3 South West Sydney Clinical Campuses, School of Clinical Medicine, University of New South Wales, Sydney, NSW Australia; 4 Orthopaedic Department, , Liverpool Hospital, SWSLHD, Liverpool, NSW Australia; 5 Physiotherapy Department, The University of Melbourne, Melbourne, NSW Australia; 6 Physiotherapy Department, Epworth Healthcare, Melbourne, VIC Australia; 7 Physiotherapy Department, Liverpool Hospital, SWSLHD, Liverpool, NSW Australia

**Keywords:** Minimally clinically important difference, Patient activation measure, Cultural diversity

## Abstract

**Purpose:**

To estimate the Minimally Clinically Important Difference (MCID) for the Patient Activation Measure (PAM) in a culturally diverse sample of people with chronic disease.

**Methods:**

This study was embedded in a larger cluster randomised trial exploring chronic disease management interventions in a culturally diverse sample of 254 adults. Patients were consecutively recruited across 16 outpatient clinics in Sydney, Australia and completed a range of outcomes related to the trial, including the Patient Activation Measure at recruitment and 6 months later. For the current study, the MCID for the PAM was estimated using two distribution methods and four anchor-based methods: mean change difference, and from the receiver operating characteristic curve, the Euclidean and Farra methods, and the Youden Index. A modified self-reported global impression of change, aligned to key elements of the PAM, was used as the external criterion and scores were dichotomised into ‘improved’ and ‘not improved.’ Therefore, the results are likely to overestimate the MCID.

**Results:**

228 participants completed pre- and post-assessments; 51% in the improved group and 49% in the not improved group. Anchor-based methods produced MCIDs between 4 and 7.9, with a value of 4.7 from the preferred Youden method. Distribution-based methods produced MCIDs ranging from 0.26 (standardised effect size) to 5.88 PAM points.

**Conclusion:**

Different methods of calculating the PAM for MCID provided different results. Using the preferred Youden Index method, an MCID of 4.7 PAM points was identified as clinically meaningful in this culturally diverse chronic disease cohort. Wider estimates from other anchor-based methods (4.0 to 7.9) are reported to illustrate methodological variability.

**Trial registration:**

Study data were collected during a two-phase hybrid cluster randomised controlled trial, approved by the South Western Sydney Local Health District Human Research Ethics Committee (2021/ETH12279) and registered prospectively on the Australian and New Zealand Clinical Trials Registry (ACTRN12622000697785).

## Abstract (plain language summary)

Patient activation empowers individuals to actively manage their health and healthcare. Greater activation helps patients to follow treatment plans and improve their health outcomes and experiences, potentially lowering their healthcare costs and improving their quality of life. The Patient Activation Measure (PAM) is a questionnaire used to assess a patient’s ability to self-manage their health-related condition. However, the amount of improvement in a person’s score on the PAM that corresponds to a meaningful improvement in their condition, known as the minimal clinically important difference (MCID), is relatively unexplored, especially in patients from culturally diverse backgrounds. This is a significant issue for clinicians and patients alike because an MCID number is influenced by the environment in which it was collected, reflecting the patients who completed it, their backgrounds and conditions, and other clinical factors. This study documents the range of MCIDs for the PAM that were calculated using different methods in a culturally diverse group of patients who received treatment in outpatient clinics for a range of chronic diseases. We found that improvements in patient activation of more than 4.7 in the PAM can be considered clinically relevant in the context of a culturally diverse group of patients with chronic conditions.

## Introduction

Patient activation is a critical concept in healthcare that encompasses a patient’s knowledge, skills and confidence in managing their health and healthcare decisions [1]. The Patient Activation Measure (PAM) is a validated 13-item psychometric questionnaire [2] used to assess a patient’s ability to self-manage across 4 domains: skill, knowledge, beliefs and behaviours. Responses are scored on a five-point Likert scale, transformed to achieve a standardised metric from 0 (lowest) to 100 (highest), then classified into one of four levels of activation. Lower levels of activation represent a patient’s limited engagement in their health, potentially arising from limited knowledge and understanding of their condition [3]. Conversely, higher levels of activation are associated with fuller engagement and responsibility (self-management) for their health condition, likely to reflect a patient’s greater levels of knowledge and understanding in self-management and how to navigate the health care system [4]. Therefore, interventions targeting patient activation have significant potential to maximise health outcomes by fostering improved self-management behaviours [5], making the PAM a valuable tool for both clinical practice and research across various chronic conditions [1, 4, 6, 7].

Importantly, and from a quality of life perspective, there is evidence that ‘patient activation’ is a significant predictor of health behaviours that reflect quality of life, such as medication adherence, and costly utilisation of health services, including hospitalisation and attendances at emergency departments [1, 8–10]. Specifically, high PAM scores have been shown to correspond to positive health outcomes and healthcare utilisation patterns across multiple studies [1, 8–10], and higher self-reported health status, higher levels of physical activity and less symptom burden for multiple chronic diseases. For instance, in a cohort of > 25,000 patients attending 35 primary care clinics in the United States, a 10-point increase in patient activation (as measured by the 13-item PAM) was associated with a 1% lower predicted probability of emergency department visits, obesity, or smoking, and a 1% higher probability of receiving breast cancer screening or having key clinical indicators (such as diabetes and cholesterol markers) in the normal range [6]. Similarly, a higher PAM (using the 22-item scale) has been shown to predict healthier cholesterol profiles and fewer all-cause hospital discharges (a key indicator of lower overall healthcare utilisation) among nearly 1,900 adults with diabetes [11]. Likewise, in a sample of > 3000 patients with chronic kidney disease, lower activation levels (13-item PAM) were associated with a greater symptom burden and poorer health-related quality of life outcomes, measured with the EuroQol 5 dimension questionnaire (EQ-5D) across various stages of disease and treatment modalities [12]. Collectively, these findings potentially demonstrate that at the population level, modest improvements in activation can yield substantial public health benefits for the millions living with chronic disease, and importantly, the relationship between patient activation and quality of life extends across multiple chronic conditions.

Beyond the associations between patient activation and quality of life, interventions that target activation in chronic disease have achieved from small to large effects in health markers that reflect improved physiological (blood pressure, HbA1c, cholesterol, weight), psychological and behavioural (self-efficacy and health-related quality of life) outcomes over time [6, 10, 13]. Therefore, the PAM has been used to provide clinical information as a single measure to tailor treatment towards activation levels, and/or as a pre- versus post-measure to assess the effectiveness of interventions to develop and enhance patient activation and self-management [3, 14] that influence quality of life. However, while some data demonstrate patients with higher activation levels experience fewer hospitalisations, reduced emergency department visits [15] and reduced healthcare costs (2–3% reduction with a single point increase in the PAM [16]), there are limited data regarding how much improvement in patient activation represents a clinically meaningful difference to a patient-consumer, which is referred to as the minimal clinically important difference (MCID) [17], and especially in patients from various culturally and linguistically diverse (CALD) backgrounds.

Importantly, MCID values are different to statistical assessments of change in response to intervention [17]. Until recently, instead of a calculated MCID, authors proposed a difference of 5 points on the PAM as a minimally important difference from comparisons between groups demonstrating health-seeking behaviours and those that did not [18], or quoted 4 points [19] from previous research that did not calculate MCID [20], but instead quoted suggestions in the original derivation papers [2, 21]. Potentially, this deficit in the literature is because there is no agreed method of calculating MCID [22–24]. However, a recent study [25] estimated the MCID of the 13-item PAM to be 5.4 (95% CI: 3.4–4.7) in a cohort of patients with chronic kidney disease. Thus, there are emerging data to assist clinicians in meaningfully interpreting changes in patient activation for the PAM, but data derived from patients from CALD backgrounds is limited, which justifies an investigation of the MCID of the PAM in patients with such a background.

A full review of the merits and limitations of the different methods of calculating MCID, including consensus-based, latent class analysis, standard deviation units and smallest worthwhile effect, is beyond the scope and intention of this paper, and such discussion is covered elsewhere [22, 23, 26]. However, the concept underlying MCID is additionally complicated by the combination of the descriptors, ‘minimal’ and ‘clinically important.’ Some methods of calculating MCID (distribution methods) do not include a patient/consumer contribution and are statistically derived with a focus on ‘minimal.’ Conversely, anchor-based methods compare changes in one outcome against a reference [22, 23], such as a global rating of change score [27], which is patient-rated to determine ‘clinically important.’ Irrespective of how it is determined, a meaningful change in a health outcome to a patient/consumer might be different and potentially less than what might be ‘significant’ to a statistician or, importantly, funders of health care.

As alluded to earlier, another consideration regarding the PAM is the characteristics of the sample from which it is derived, which may influence the utility and generalisability of how it is applied [17]. Limited data were provided regarding the demographics of the convenience sample of 1515 adults from which the 22-item, and in turn, the subsequent 13-item PAM was derived [2, 21]. Specifically, no data were provided regarding the ethnocultural diversity of the participants. The description regarding the characteristics of patients with kidney disease from which the recent MCID of the PAM was derived was limited to “white British ethnicity” [25]. Since the proportion was > 90% overall [25], and ethnocultural background influences a consumer’s ability to navigate healthcare, with effects on health outcomes [28–30] and ipso facto, their quality of life, it can be argued that there remains a deficit with respect to the influence of CALD on the PAM. Therefore, if the PAM is used to assess the self-management skills of patients from various CALD backgrounds, an MCID should be derived from participants from CALD backgrounds.

Therefore, the purpose of this investigation was to determine the MCID for the PAM and specifically, from a sample of patients from diverse CALD backgrounds, using both anchor-based methods and distribution-based methods. It was nested within a larger cluster randomised trial implemented in an Australian health district with a high proportion of residents from CALD backgrounds. South Western Sydney Local Health District (SWSLHD) serves over one million residents, with > 50% born overseas and > 40% speaking a language other than English at home [31]. The trial targeted patients with chronic diseases attending several clinics and specifically, patients from CALD backgrounds, including but not limited to Vietnamese, Arabic-speaking, Assyrian and Pasifika communities [32]. Notably, many patients in such communities, reflected in data collected for the current study, reported several intercurrent diagnoses in addition to the condition for which they attended the target health clinic.

## Methods

### Design

Data were collected prospectively during a two-phase hybrid cluster randomised controlled trial of a cultural mentor program with an observational arm and a cluster RCT sample. The study, approved by the South Western Sydney Local Health District Human Research Ethics Committee (2021/ETH12279) and registered prospectively on the Australian and New Zealand Clinical Trials Registry (ACTRN12622000697785), is described in detail elsewhere [32]. The primary aim of the cluster trial was to evaluate whether participants who received culturally oriented mentorship (intervention) achieved greater changes in their self-activation for chronic disease management, measured via the PAM at 6 months post-treatment, compared to those receiving usual care (control). Thus, power was calculated for the primary outcome in the larger hybrid cluster randomised controlled trial and not for the current secondary analysis.

### Participants and procedure

Participants were drawn from 16 participating healthcare clinic clusters in public hospitals across multicultural regions of Australia. All participants were recruited at the commencement of treatment for a chronic and/or complex condition for which self-management was a key component, including diabetes, musculoskeletal, pain, rheumatology, cancer, lymphoedema, respiratory and cardiac. Participants were eligible if they were aged ≥ 18 years, commencing active treatment in a participating cluster, and self-identified with a CALD community targeted for cultural mentoring by the clinic. Exclusion criteria included a clinical diagnosis that could impair cognition and capacity to provide written informed consent. Translated participant information sheets and consent forms were provided for participants who spoke a language other than English.

Consecutive consenting participants recruited to the study completed a sociodemographic survey, the functional comorbidity index [33] and standardised assessments appropriate to the trial [32]. All outcome assessments utilised translated outcome measures and were available in English, Arabic and Vietnamese. Across the clusters, participants received either usual care determined by the treating clinical team or a combination of cultural mentoring delivered by a volunteer cultural mentor with lived experience of the chronic disease, integrated alongside usual care. Participants received treatment in face-to-face, virtual or phone consultation format for up to 6 months.

### Outcomes

#### Patient activation measure (PAM)

Participants self-reported their knowledge, skills and confidence managing their health using the PAM questionnaire, which they completed at baseline and again at 6 months on a scale (‘strongly disagree,’ ‘disagree,’ ‘agree,’ ‘strongly agree, ’ and ‘not applicable’) for each of the 13 items [2]. Responses are transformed using Insignia’s© proprietary natural algorithm to achieve a standardised metric ranging from 0 to 100 (0 = lowest activation to 100 = highest activation) and classified into one of four levels of activation (Level 1, characterised as not believing activation is important, to Level 4, characterised as taking action but requiring support in maintaining positive behaviour change). The 13-item PAM has reported high internal consistency reliability (Cronbach’s alpha: 0.8–0.9), good to excellent test-retest reliability (intraclass correlation coefficients: 0.76 and 0.98), satisfactory content and face validity, and significant correlations with measures of self-efficacy and locus of control (moderate to strong correlation coefficients) [34–36]. The MCID in a patient cohort of predominantly “white British” patients with chronic kidney disease was a mean of 5.4 points anchored against the Short Form-12 survey [25]. This study used the English, Arabic and Vietnamese versions of the PAM, which has been translated into 35 different languages [37], reflecting the preferred language and diversity of the target cohort.

#### Anchor question: global rating of change (GRC)

For anchor-based analyses of MCID, participants were also asked a question (GRC) aligned to key elements from the PAM to appraise the change in their activation over six months using a seven-point Likert scale according to ‘much,’ ‘moderately’ or ‘slightly worse’ (-3 to -1), ‘no change’ (0) to ‘slightly’, ‘moderately’ or ‘much better’ (+ 1 to + 3):

Compared with before receiving treatment with this clinic, how would you describe your knowledge, confidence and skill in managing your health?

The GRC scale, particularly the 7-point version, is a valid and widely used tool for evaluating self-reported change in clinical contexts [27] and has been applied previously for determining the minimally clinically important difference [38].

## Analyses

The current study was secondary to the primary study and not powered to investigate the MCID. Further, finite external funding prevented increasing the sample size, even if interim power analyses were conducted. Therefore, it was determined a-priori and pragmatically that certain categories for the GRC anchor would be combined [39], with implications for the resultant MCID (see next Section: Analyses of thresholds). A study biostatistician (JD) performed data analyses, independent of the implementation team and blinded to participant allocation. Sociodemographic and health profiles were descriptively summarised and analysed using R version 4.5.

### Analyses of thresholds

Acknowledging the considerable variability and potential lack of convergence inherent in different methodologies to calculate MCID, we employed both anchor-based methods and distribution-based methods [22, 23] to enhance the generalisability of our findings regarding the PAM. Anchor-based approaches utilise an external patient-centred criterion (the GRC for current data) to evaluate whether change scores (in the PAM) were clinically meaningful. Initially, Spearman’s rank correlation was performed to evaluate the association between the change in PAM scores (six-month follow-up minus baseline) and the patient’s perceived improvement determined from the GRC. Correlations were interpreted according to strong (> 0.5), moderate (0.3 to 0.5), and weak (< 0.3) [40].

Participant responses for the GRC were then dichotomised into a “*not-improved*” group (‘*much worse’* to *‘slightly better*,’ spanning Likert responses from − 3 to + 1) and an ‘*improved*’ group (‘*moderately better’* and *‘much better*’ combining responses + 2 and + 3). This pre-specified, conservative grouping was chosen to reduce potential boundary-level contamination at the 0/+1 interface in a CALD diverse cohort, where acquiescence/“willingness to please” and related social-desirability response styles are more likely to operate at points of evaluative ambiguity (e.g., nudging “no change” toward “slightly better”) [41, 42]. We recognise that this allocation departs from the conventional use of “slightly better” to represent minimal improvement and may increase the resulting threshold relative to +1-based definitions. Based on this dichotomisation, the MCID of PAM was calculated using four anchor-based methods after a receiver operating characteristic (ROC) curve, with ‘*improved’* as the reference group, was plotted and the PAM change score from baseline to follow-up as the predictor.

In the ROC curve analysis, we first determined the optimal cutoff score and the area under the curve (AUC) with accompanying 95% confidence intervals. An AUC of 0.75 or higher has been suggested as a threshold for clinical utility [43]. An MCID was calculated using the Youden Index, which corresponds to the point at which the sum of the sensitivity and specificity is maximised. Second, the Euclidean method was calculated, which corresponds to the point closest to the top left of the curve. Third, the Farra method was calculated, which corresponds to the point that minimises the difference in sensitivity and specificity. Finally, the fourth estimate was derived by calculating the difference in PAM change between the improved and not-improved groups. We determined that we would use the Youden method as the primary approach to calculate the MCID because it maximises both sensitivity and specificity to distinguish participants who might experience a meaningful improvement from those who did not [23]. Alternative anchor-based methods were included as supporting analyses.

Distribution-based methods rely on the statistical characteristics of a sample and analyse the capacity of the instrument to detect change in general [23]. Consistent with methodological recommendations [23], these indices were not used to define the MCID. Instead, they are presented as supporting information to characterise measurement variability and the magnitude of change. These were estimated from: (1) 50% of the standard deviation of the baseline PAM score, and (2) pre-to-post PAM difference divided by the SD.

## Results

Two hundred and fifty-four patients were recruited overall. Twenty-six patients did not have a PAM or GRC score at follow-up and were excluded from the MCID analyses. All participants were analysed together for MCID calculations, independent of group allocation. Participant characteristics are summarised in Table 1.

**Table 1:** Participant characteristics

	Cohort (*n* = 254)	Analysis cohort (*n* = 228)
Age, years, mean (SD)	61.3 (12.6)	61 (12.5)
Gender, female, n (%)	143 (56)	133 (58)
COB, n (%) Australia or NZ Iraq Lebanon Vietnam Other*	18 (7) 76 (30) 74 (29) 24 (9) 62 (24)	16 (7) 72 (32) 63 (28) 24 (11) 53 (23)
Years lived in Australia, mean (SD)	25.8 (16.8)	25.4 (16.4)
Ethnocultural Identification, n (%) Arab Assyrian or Chaldean Pacific Island culture Vietnamese Mandaean Other Middle Eastern cultures Other^	103 (41) 50 (20) 37 (15) 24 (9) 23 (9) 11 (4) 6 (2)	89 (39) 50 (22) 28 (12) 24 (11) 22 (10) 10 (4) 5 (2)
Interpreter required for communication, n (%)	155 (61)	140 (61)
Preferred Language, n (%) English Arabic Assyrian Vietnamese Other **~**	30 (12) 139 (55) 30 (12) 24 (9) 31 (12)	20 (9) 124 (54) 29 (13) 24 (11) 31 (14)
Highest education attainment, n (%) No schooling Elementary or junior high Senior high Post-school trade, certificate or degree	22 (8) 104 (41) 65 (26) 63 (25)	16 (7) 96 (42) 58 (25) 58 (25)
Functional comorbidity index, mean (SD)	5 (3.1)	4.9 (3.1)
Baseline PAM, Mean (SD)	55.3 (12.3)	55 (11.8)

COB, country of birth; *Denotes 11 other countries of birth; ^relates to four other ethnocultural identities; ~ relates to nine other languages

### Correlation of change in PAM with perceived change in knowledge, skill, and confidence

**Fig. 1:**
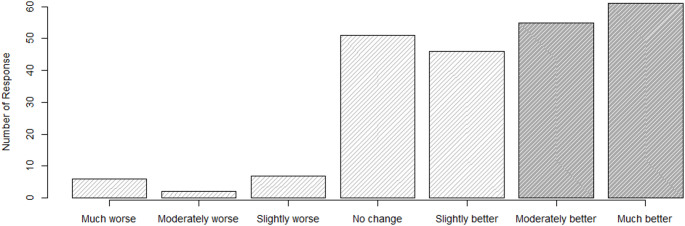
Responses to GRC (Anchor Question)

The distribution of responses for the anchor question is observed in Fig. 1 and demonstrates a majority of patients reporting improvements in response to GRC regarding their knowledge, confidence and skill in managing their condition.

**Fig. 2:**
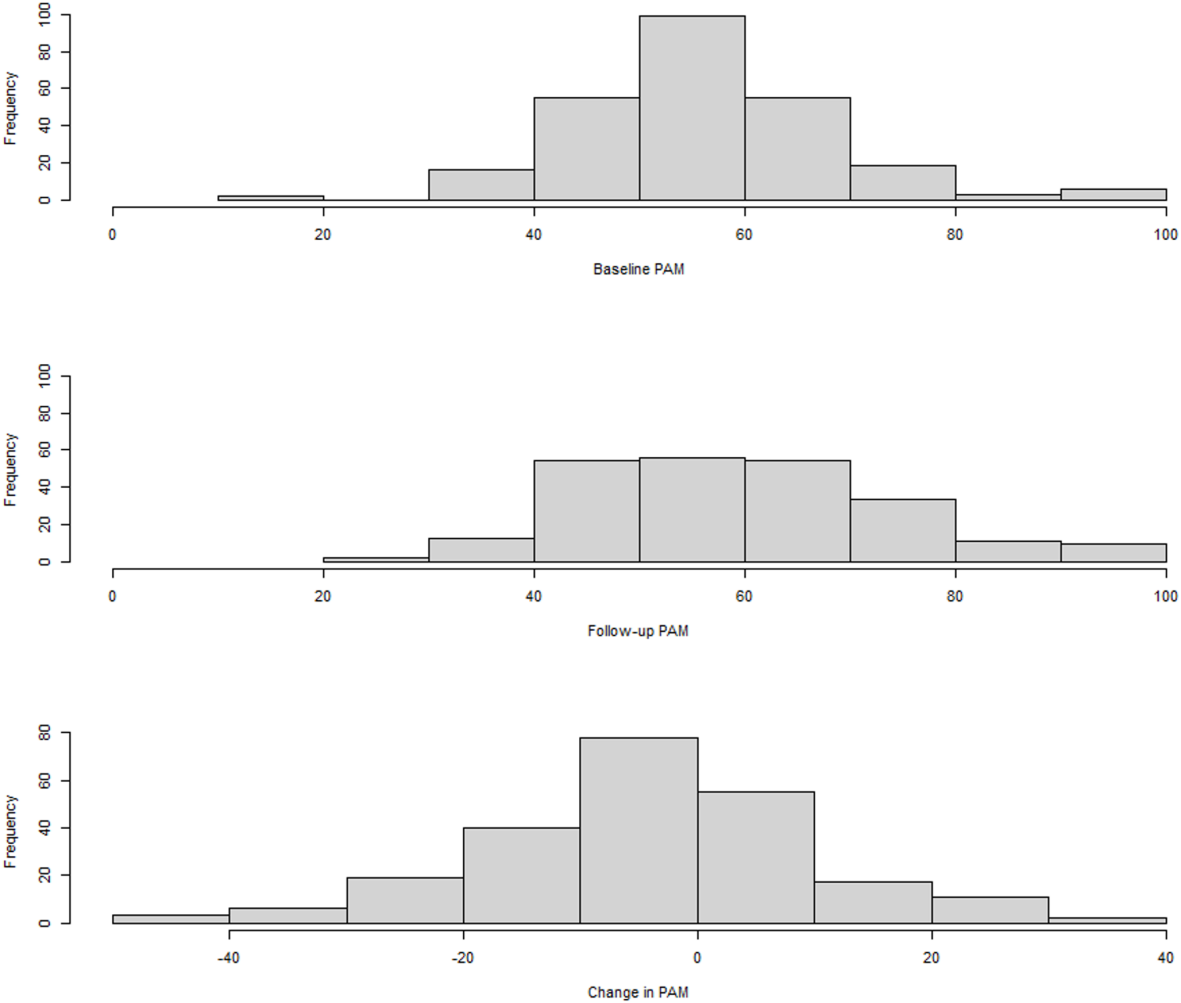
PAM scores at baseline, follow-up and overall change scores

Figure 2 illustrates the frequency distribution for PAM scores for all participants. The overall average PAM score at baseline was 55.3 (SD 12.3) and at follow-up was 59.1 (14.4) for an overall average increase of 3.8 (14.7). The Spearman’s correlation between PAM and the GRC regarding perceived change in knowledge, skill and confidence was 0.302, indicating a borderline-moderate positive relationship (*p* < 0.001) [44].

### MCID estimation from anchor methods

**Fig. 3:**
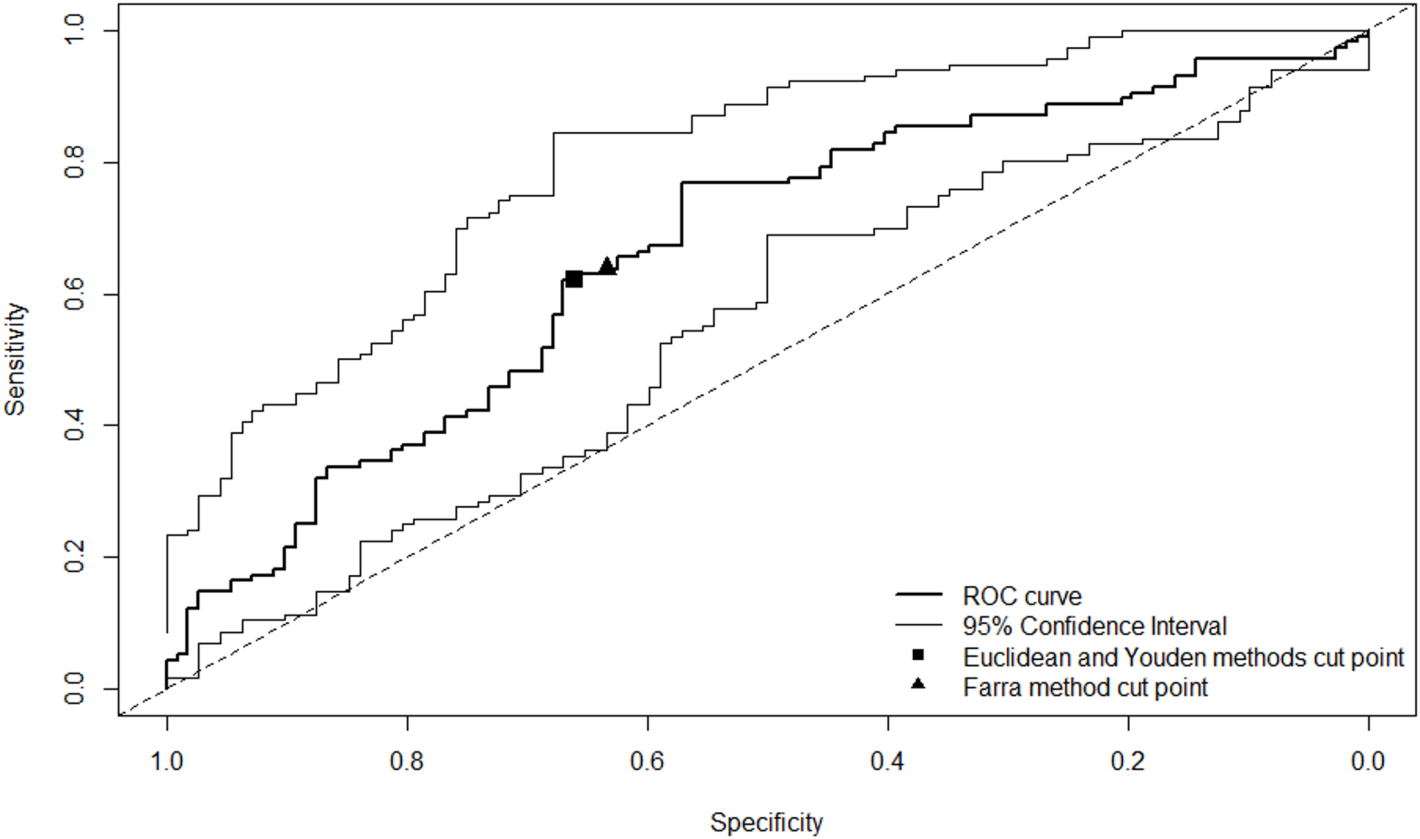
Area under the curve for dichotomised outcomes versus PAM

A ROC curve (Fig. 3) was calculated for the dichotomised anchor-based groups of “*not-improved*” (n = 112) and “*improved*” (n = 116) with PAM change as the outcome, with a resultant AUC of 0.66 (95% CI 0.59–0.73). The Youden and Euclidean methods estimated an MCID of 4.7, with a sensitivity of 0.62 (95% CI 56–79) and specificity of 0.66 (95% CI 53–74), respectively. The Farra method estimated an MCID of 4.0, with a sensitivity of 0.64 (95% CI 56–70) and specificity of 0.63 (95% CI 57–70). For patients who improved (*‘moderately’* to *‘much better’)*, the mean baseline PAM was 57.8 (SD 13.4) and at follow-up was 65.6 (SD 13.9) for a mean PAM change of 7.8 (SD 15.1). For those in the not-improved group (*‘much worse’* to *‘slightly better’)*, the mean baseline PAM was 52.1 (SD 8.9) and at follow-up was 52.0 (SD 11.1) for a mean PAM change of -0.1 (SD 13.3). Thus, the MCID based on a difference in the change between groups was + 7.9 (95% CI 4.2–11.6).

### MCID estimation based on distribution methods

The SD of PAM at baseline was 11.8, yielding an estimate of MCID for PAM of 5.88 based on 50% of the baseline SD. The mean PAM at baseline for the cohort was 55 (11.8), and 58.9 (14.3) at follow-up, resulting in a mean change in the difference of 3.9 with an SD of 14.7. Thus, the MCID based on the ratio of the mean difference and the SD for the difference (3.9/14.7) gives 0.26, representing a small standardised effect size.

## Discussion

The purpose of the current study was to provide data regarding the MCID of the PAM for clinicians to assess patient activation in health care settings, with implications for their quality of life. Consistent with previous research, they demonstrate that the MCID is dependent upon the method used to calculate it [17, 22]. Clinicians should be aware of such variability if they are to interpret an individual’s activation status from the PAM before delivering an intervention, or their response to an intervention afterwards, when comparing pre- versus post-intervention PAM scores, especially if the purpose is to change behaviour to improve quality of life.

Our data, derived from a sample of patients from various CALD backgrounds with a range of intercurrent chronic conditions attending a clinic specific to one of their conditions (Table 1), suggest that a PAM change of 4.7 from the Youden method might be interpreted as clinically meaningful. This estimate aligns closely with earlier recommendations of 4 to 5, which were not statistically derived [2, 18, 21], and is also comparable to a recent statistically derived MCID of 4.5 (3.4–7.4), identified using the SF-12 questionnaire as the anchor in patients with chronic kidney disease [25]. Nonetheless, interpretation of our results and their application to clinical practice should be made with reference to the specific dichotomisation strategy used for our global rating of change anchor and sample characteristics, since they can influence MCID, per later discussion.

Our results complement recently derived MCID data for the PAM in a sample of patients of “white British ethnicity”, as reported by Wilkinson et al. [25], and extend this work by sampling a cohort of patients from CALD backgrounds. Unlike Wilkinson et al., who excluded participants without sufficient English proficiency to provide informed consent, our study included patients regardless of English language ability through translated materials and professional interpreting services. Given that subjective transition ratings can behave differently across cultural and linguistic groups [42] and that anchor credibility is essential for valid MCID estimation, differences between studies in anchor use and sample composition are likely to influence MCID values [39]. To mitigate social desirability and response-style bias [46] observed more frequently in collectivist and hierarchical cultural contexts, including acquiescence and endpoint responding that can inflate ‘slight improvement’ responses, we prespecified a more conservative responder definition [41, 42, 45]. Specifically, we classified + 2 and + 3 responses as ‘improved’ and grouped − 3 to + 1 as ‘not improved’. This decision is consistent with evidence that response styles vary systematically with culture (e.g. power distance, collectivism) and that social desirability structures differ across multi-ethnic samples, potentially affecting how patients use the lower end of improvement categories [41, 42, 45]. In contrast, Wilkinson et al. treated both ‘no change’ (0) and ‘slightly better’ (+ 1) as improved, reasoning that stability in progressive kidney disease represented a clinically meaningful outcome [25]. Such a more inclusive definition of improvement would be expected to yield a lower MCID threshold, whereas our more conservative definition is likely to produce a higher estimate, underscoring the need to interpret MCID values in light of anchor design, grouping rules, and population context [39].

Limited data were provided regarding the CALD characteristics of the sample from which the original 22-item and subsequent 13-item PAM used for the current study were derived [2, 21], other than race (“white, black and other”), which is not consistent with contemporary CALD descriptions that emphasise social and cultural self-identification, rather than biological, visible or country of origin differences [47, 48]. The current study, however, successfully used the PAM to survey participants from various CALD backgrounds regarding their self-management skills, likely because we used validated translated versions. Given that patients from CALD backgrounds are known to experience significant barriers navigating health care settings for reasons that extend beyond language literacy [29, 30], a tool like the PAM with a demonstrated utility in CALD communities will be helpful for clinicians and researchers surveying participants from similar CALD groups to the current study.

In combination with widely applied and validated outcomes to measure quality of life, such as the EQ-5D [49], an MCID for the PAM will facilitate clinicians’ understanding of whether specific changes in a patient’s activation have translated to improved outcomes and quality-of-life changes. However, there could be a ‘lag’ effect associated with patient activation and quality of life, especially in the context of interventions for chronic disease, when changes in patient activation would likely precede changes in health status. This was demonstrated in an investigation of men with prostate cancer undergoing a community lifestyle intervention, when significant improvements in PAM scores at 3 months that were maintained at 6 months did not correspond to significant changes in quality of life at the same epochs, as measured by the EQ-5D [50]. Nevertheless, in general, and consistent with previous discussion about the relationship between patient activation and quality of life, the authors observed that across intervention modalities, lower activation levels were associated with higher symptom burden and reduced HRQoL. However, while illustrating a relationship between the PAM and quality of life, assessments such as the EQ-5D, their data reinforce that the PAM and EQ-5D measure different constructs.

### Limitations

It is accepted that the categories included in the ‘*slightly improved’* and *‘not improved’* groups are not necessarily consistent with an assessment of a ‘minimal’ difference. Our category grouping, taken to mitigate suspected contamination at the 0/+1 boundary, likely raised the average change among those classified as ‘*improved’* and lowered the average change for the ‘*not-improved’* group, biasing the MCID towards a higher value. Readers should consider this potential upward bias when comparing our threshold with estimates derived from + 1-based definitions.

Potentially, our data might only be valid for the CALD groups surveyed (Table 1). However, we used translated versions of the PAM, which might be considered the equivalent of ‘standardising’ participant responses regardless of ethnocultural background. Since Insignia’s© proprietary algorithm does not differentiate the language of the PAM as it was collected, it is unclear whether the observed MCID can be generalised from this study to other translated versions of the PAM in other CALD groups.

It is unclear whether the MCIDs observed here would differ if the outcomes were measured at different time points (e.g. at 1-year post-intervention compared with 6 months for our data, or 20 weeks as recently reported [25]). Change scores are likely to vary across different epochs, and according to the patient’s particular chronic condition and disease stage, given that different conditions and stages have different levels of stability or rates of deterioration. In the current study, the matched cluster randomisation underpinning the larger trial might have minimised differences between control and intervention cohorts in the patient sample. However, the study design, while matching for a range of chronic and/or complex conditions, including diabetes, musculoskeletal, pain, rheumatology, cancer, lymphoedema, respiratory and cardiac conditions, did not control for the stage of a patient’s disease.

Though significant, the borderline-moderate Spearman’s correlation of 0.302 between the PAM and participants’ self-assessment on the GRC suggests there are differences in the constructs they are assessing. Consistent with this, the ROC analysis yielded a AUC below our a-priori clinical utility threshold of 0.75, with corresponding sensitivity/specificity estimates in the 0.6–0.7 range. These metrics indicate limited discrimination of the anchor in this population and should temper inferences about individual-level classification. Potential contributions include construct non-equivalence between PAM and the global change anchor. Using a conceptually aligned, multi-dimensional anchor may improve construct compatibility [25]. Cultural and linguistic diversity within the cohort, hitherto argued as a strength of the study and a point of difference to recent research [25], might have contributed to varied interpretation of question items, independent of language proficiency [51]. Alternatively, asking participants to recall changes in their knowledge, skills and confidence over a 6 month period introduces recall bias. Together, these factors may have attenuated the observed association and reduced the anchor’s discriminative performance in this cohort. In light of the above limitations, our MCID estimates should be regarded as context-specific to a CALD population and to an anchor whose performance was modest in this dataset.

### Summary

The current study provides data for the MCID for the PAM derived from a CALD sample with a range of chronic conditions. Using translated PAM instruments and a prespecified conservative responder definition, the primary anchor-based MCID (Youden) was 4.7 PAM points. Given this grouping, the estimate may be upwardly biased relative to conventional + 1-based definitions and should be interpreted as context-specific, rather than universal. Consistent with previous research, supplementary MCID estimates varied by approach (anchor or distribution-based), with supplementary anchor-based values ranging from 4 to 7.9. These results are broadly consistent with previous non-statistical estimates of approximately 4 to 5, and a recent estimate of a mean PAM change in a cohort of patients from a predominantly non-CALD background of 5.4 (3.4–7.4) [25]. Finally, because all data for CALD participants were collected using validated translations, our findings do not support substituting non-translated PAM forms in similar cohorts.

## Data Availability

The data that support the findings of this study are available from the corresponding author upon reasonable request.
